# Supplementary figures and images for: The insertion of a mitochondrial selfish element into the nuclear genome and its consequences
Source: Ecol Evol. 2020 Aug 31;10(20):11117–32. doi: 10.1002/ece3.6749 (PMC7593156; doi:10.1002/ece3.6749)

A)

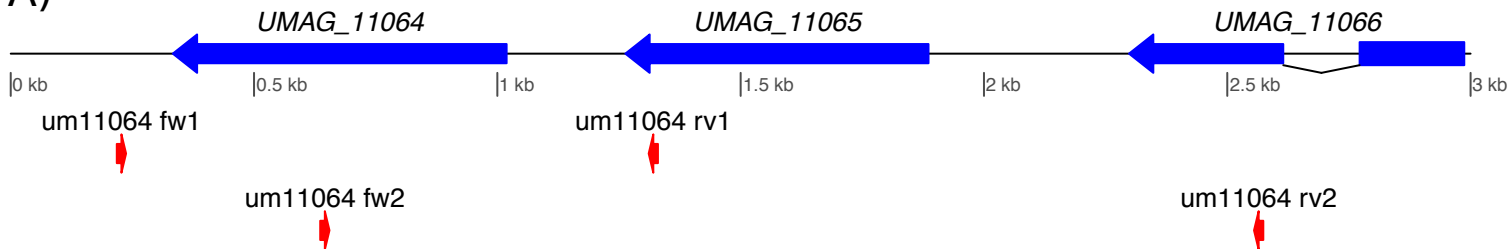

B)

fw1-rv1

fw1-rv2

fw2-rv2

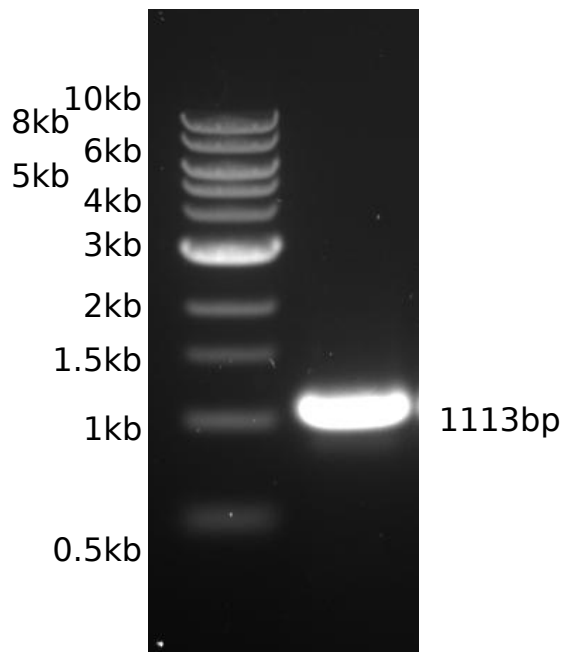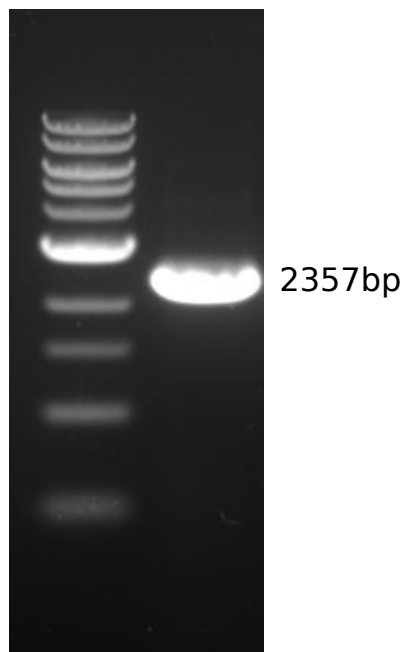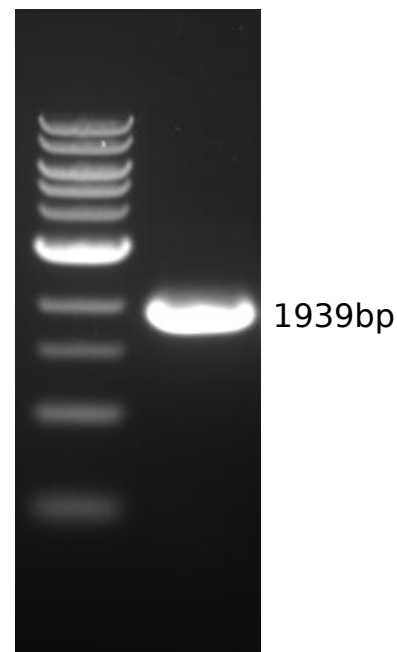

Supplement: Supplementary file 1 — Figure S1 [file ECE3-10-11117-s001.pdf]

**A)**

SG200

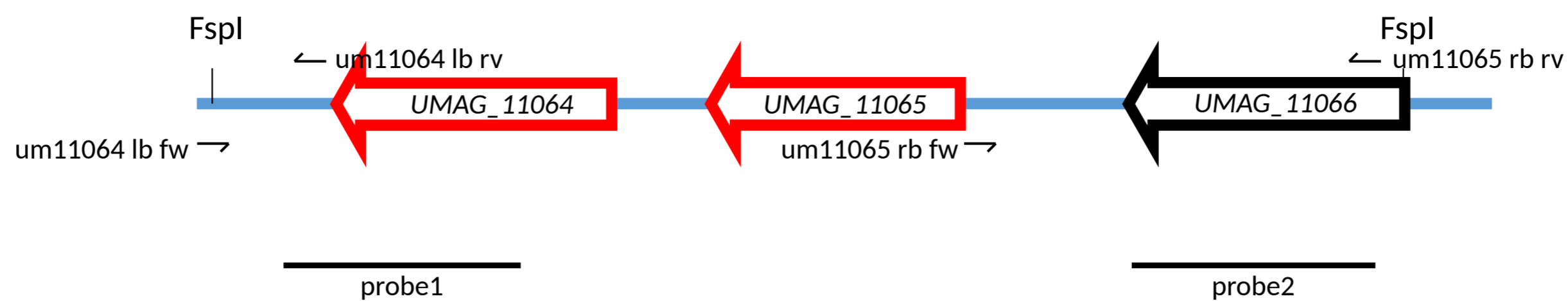SG200 $\Delta$ 11064 $\Delta$ 11065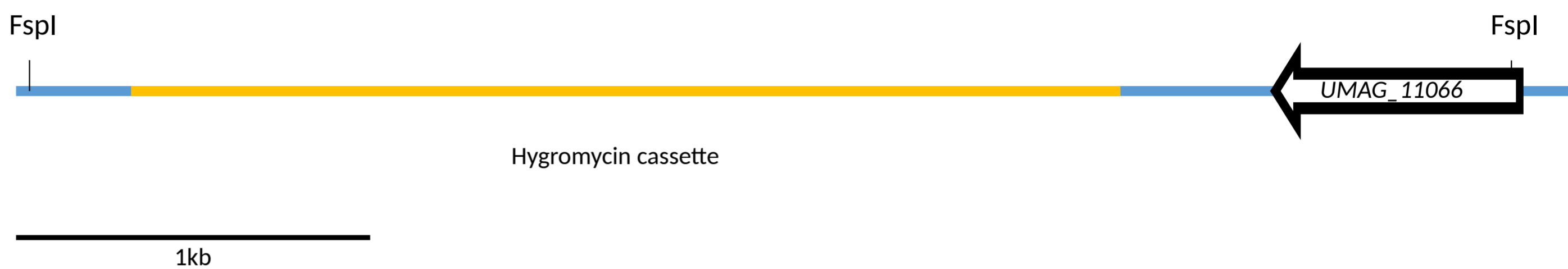**B)**

DNA ladder (kb)

SG200

SG200 $\Delta$ 11064 $\Delta$ 11065

10

8

6

5

4

3

2

4.19 kb

2.94 kb

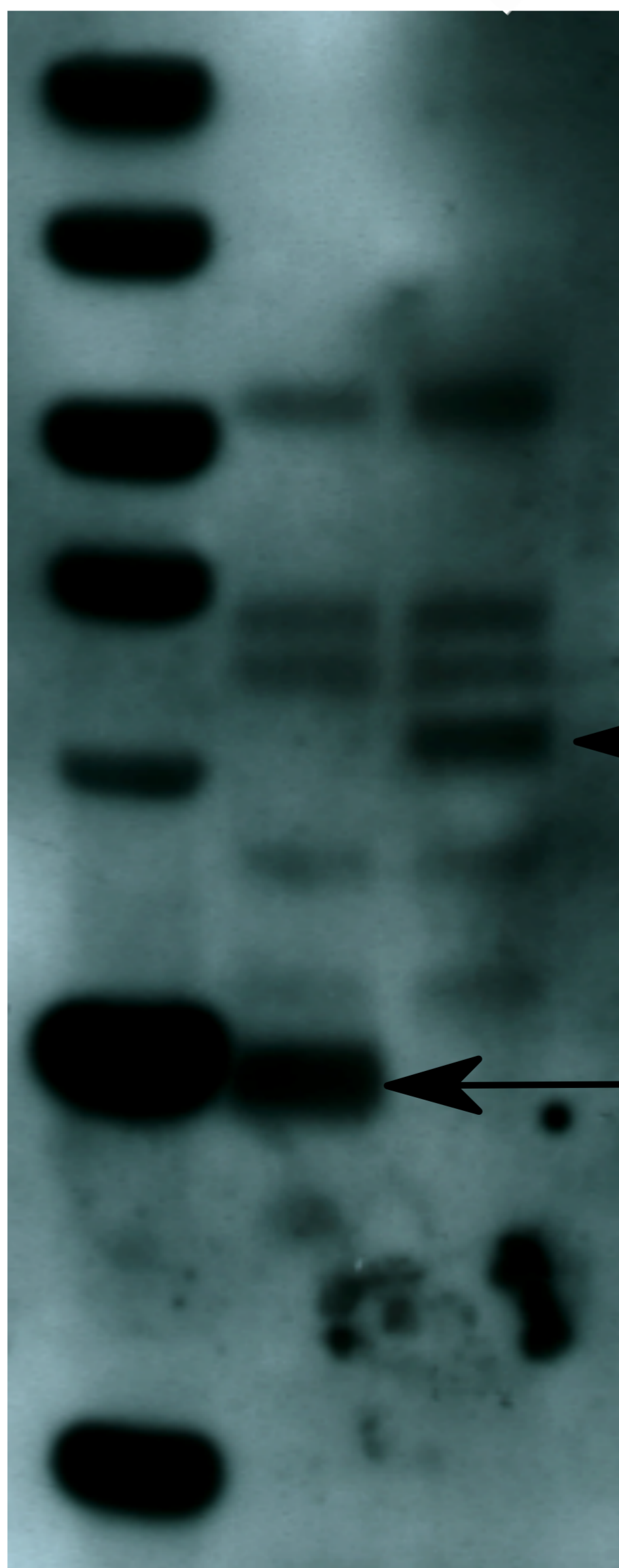

Supplement: Supplementary file 3 — Figure S3 [file ECE3-10-11117-s003.pdf]
