# Supplementary material for: The insertion of a mitochondrial selfish element into the nuclear genome and its consequences
Source: Ecol Evol. 2020 Aug 31;10(20):11117–32. doi: 10.1002/ece3.6749 (PMC7593156; doi:10.1002/ece3.6749)
Supplement: Supplementary file 2 — Figure S2 [file ECE3-10-11117-s002.pdf]

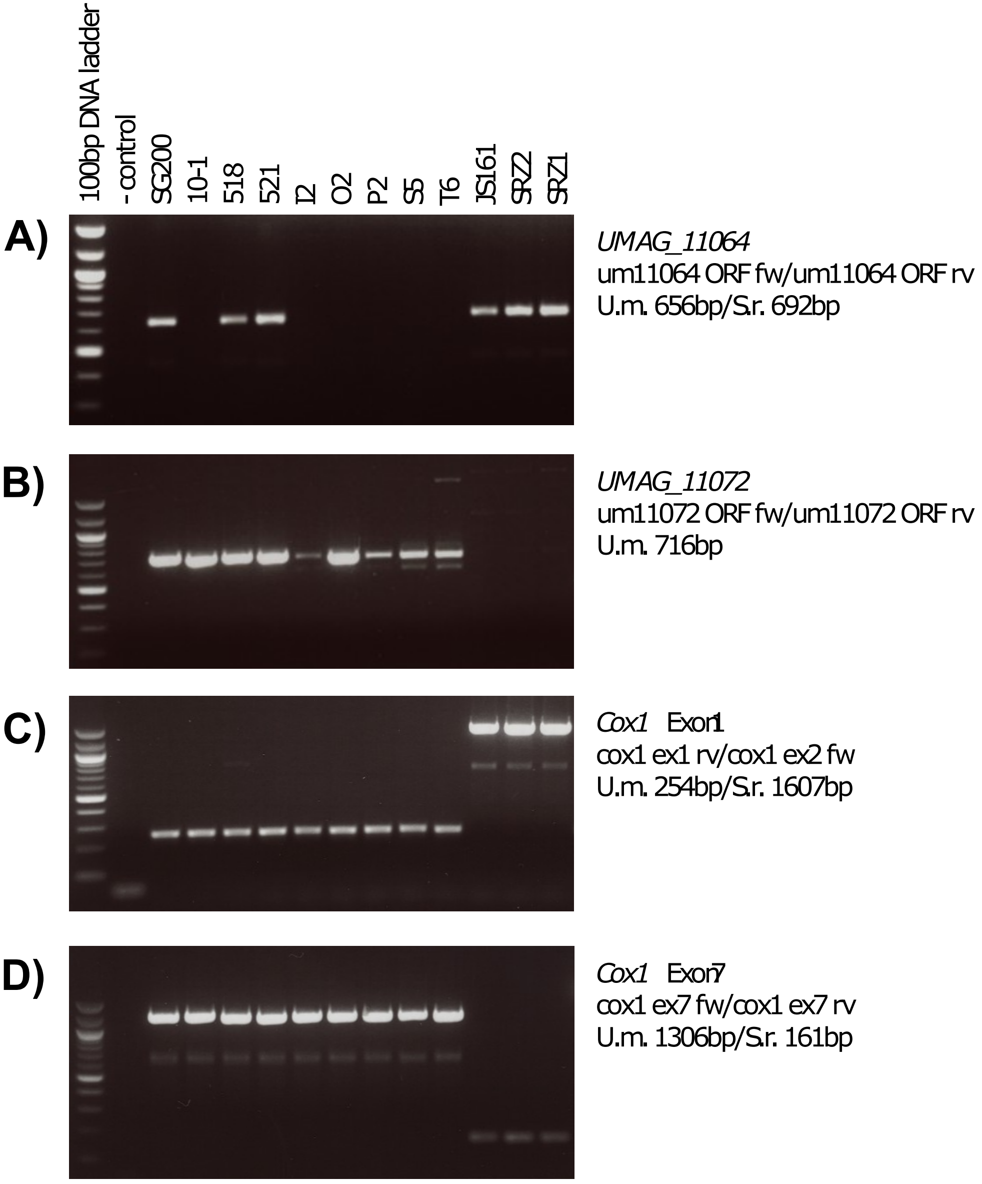

E)

Region	Strain											
	U. maydis									*S. reilianum*		
	SG200	10-1	518	521	I2	O2	P2	S5	T6	JS161	SRZ1	SRZ2
UMAG_11064 ORF	+	-	+	+	-	-	-	-	-	+	+	+
UMAG_11072 ORF	+	+	+	+	+	+	+	+	+	(not tested)		
Cox1 Exon 1+2	254	254	254	254	254	254	254	254	254	1607	1607	1607
Cox1 Exon 7	1306	1306	1306	1306	1306	1306	1306	1306	1306	161	161	161
